# Detection of selection signatures in dairy and beef cattle using high-density genomic information

**DOI:** 10.1186/s12711-015-0127-3

**Published:** 2015-06-19

**Authors:** Fuping Zhao, Sinead McParland, Francis Kearney, Lixin Du, Donagh P Berry

**Affiliations:** National Center for Molecular Genetics and Breeding of Animal, Institute of Animal Sciences, Chinese Academy of Agricultural Sciences, Beijing, 100193 China; Animal and Grassland Research and Innovation Centre, Teagasc, Moorpark, Co., Cork, Ireland; Irish Cattle Breeding Federation, Highfield House, Bandon, Co., Cork, Ireland

## Abstract

**Background:**

Artificial selection for economically important traits in cattle is expected to have left distinctive selection signatures on the genome. Access to high-density genotypes facilitates the accurate identification of genomic regions that have undergone positive selection. These findings help to better elucidate the mechanisms of selection and to identify candidate genes of interest to breeding programs.

**Results:**

Information on 705 243 autosomal single nucleotide polymorphisms (SNPs) in 3122 dairy and beef male animals from seven cattle breeds (Angus, Belgian Blue, Charolais, Hereford, Holstein-Friesian, Limousin and Simmental) were used to detect selection signatures by applying two complementary methods, integrated haplotype score (iHS) and global fixation index (F_ST_). To control for false positive results, we used false discovery rate (FDR) adjustment to calculate adjusted iHS within each breed and the genome-wide significance level was about 0.003. Using the iHS method, 83, 92, 91, 101, 85, 101 and 86 significant genomic regions were detected for Angus, Belgian Blue, Charolais, Hereford, Holstein-Friesian, Limousin and Simmental cattle, respectively. None of these regions was common to all seven breeds. Using the F_ST_ approach, 704 individual SNPs were detected across breeds. Annotation of the regions of the genome that showed selection signatures revealed several interesting candidate genes i.e. *DGAT1, ABCG2*, *MSTN, CAPN3, FABP3*, *CHCHD7*, *PLAG1*, *JAZF1*, *PRKG2*, *ACTC1*, *TBC1D1*, *GHR*, *BMP2*, *TSG1*, *LYN*, *KIT* and *MC1R* that play a role in milk production, reproduction, body size, muscle formation or coat color. Fifty-seven common candidate genes were found by both the iHS and global F_ST_ methods across the seven breeds. Moreover, many novel genomic regions and genes were detected within the regions that showed selection signatures; for some candidate genes, signatures of positive selection exist in the human genome. Multilevel bioinformatic analyses of the detected candidate genes suggested that the PPAR pathway may have been subjected to positive selection.

**Conclusions:**

This study provides a high-resolution bovine genomic map of positive selection signatures that are either specific to one breed or common to a subset of the seven breeds analyzed. Our results will contribute to the detection of functional candidate genes that have undergone positive selection in future studies.

**Electronic supplementary material:**

The online version of this article (doi:10.1186/s12711-015-0127-3) contains supplementary material, which is available to authorized users.

## Introduction

Artificial selection in cattle has resulted in divergent breeds that are specialized for either milk or meat production or raised as dual-purpose breeds. Such selection strategies are likely to have imposed selection pressures on particular regions of the genome that control these traits as well as other important animal characteristics such as disease resistance or general immune competence. Under positive selection pressure, the frequency of favorable alleles in the genome will rapidly increase. If intensive selection pressure occurred only over a few generations, it is unlikely that recombination had an impact on haplotype structure, and thus it resulted in (extended) linkage disequilibrium (LD) patterns between the mutation and neighboring loci [1]. Analysis of these selection signatures can reveal genomic regions of interest for selection and provide insights into the mechanisms of evolution [2, 3].

Various statistical approaches have been proposed for the detection of selection signatures. Such tests include Tajima’s D-statistic [4], Fay and Wu’s H-statistic [5], extended haplotype homozygosity (EHH) [1], integrated haplotype score (iHS) [6], the Ka/Ks test [7], and the McDonald and Kreitman test [8]. The EHH test is particularly useful to detect signatures of positive selection within a population using single nucleotide polymorphism (SNP) data [9–11]. This method that was first developed by Sabeti *et al.* [1] exploits knowledge on the relationship between the frequency of an allele and the measures of LD with neighboring alleles. An EHH is defined as the probability that two randomly chosen chromosomes that carry the core haplotype of interest are identical by descent for the entire interval between the core region and a certain locus [1]. To overcome the influence of heterogeneous recombination rates across the genome, Voight *et al.* [6] developed the iHS approach, which is an extension of the EHH method and is based on the comparison of EHH between derived and ancestral alleles within a population. The iHS achieves maximal power when a selected allele segregates at intermediate frequencies in the population.

An alternative approach to the detection of selection signatures is based on the measure of population differentiation due to locus-specific allele frequencies between populations, which is quantified using the F_ST_ statistic [12]. The fixation index, F_ST_ was first defined by Wright [13] to quantify the degree of genetic differentiation among populations based on differences in allele frequencies. F_ST_ provides information on the genomic variation at a locus among populations relative to that within populations. Thus, F_ST_ is also a test for evidence of selection i.e. high F_ST_ values indicate local positive adaptation while low F_ST_ values suggest negative or neutral selection [14].

Both iHS and F_ST_ statistics are useful to detect selection signatures [15]. Previous analyses suggested that they are largely complementary; iHS has good power to detect selection signatures within breeds, while global F_ST_ is useful to detect selection signatures (*i.e.*, loci that were differentially fixed in different breeds) across breeds [16]. Global F_ST_ is also used to determine how divergent selection has impacted the genome of these breeds. The objective of our study was to detect signatures of selection using a large dataset of beef and dairy cattle with high-density SNP genotyping data. Potential biological functions of the genes that are present in the identified selection signatures were also examined using multi-level bioinformatic analyses.

## Methods

### Ethics statement

Animal Care and Use Committee approval was not obtained for this study because all the data used were from the pre-existing database infrastructure operated by the Irish Cattle Breeding Federation (ICBF, Bandon, Co. Cork, Ireland).

### Genotypes

Illumina (http://www.illumina.com) high-density genotypes (777 962 SNPs) were available on 3122 dairy and beef bulls; all animals had a genotype call rate of at least 95 %. The number of bulls per breed was 269, 196, 710, 234, 719, 730, and 264 for Angus, Belgian Blue, Charolais, Hereford, Holstein-Friesian, Limousin and Simmental, respectively. Mendelian inconsistencies among autosomal genotypes were used to validate animal identification through parentage assessment but also to discard 2816 reportedly autosomal SNPs that did not adhere to Mendelian inheritance patterns. An additional 11 654 autosomal SNPs with GenTrain scores less than 0.55 (*i.e.*, a measure of genotype call quality) and a call rate less than greater than 90 % were also discarded as well as 29 939 SNPs that were monomorphic across all breeds or for which the position on the genome was unknown. The UMD3.1 genome build was used. Missing genotypes were imputed and genotypes were phased using Beagle Version 3.1.0 (http://faculty.washington.edu/browning/beagle/beagle.html) [17, 18]. After quality control, 705 243 SNPs were available with a mean distance of 3.56 kb between adjacent SNPs [See Additional file 1: Table S1].

### Calculation of inbreeding coefficients

The pedigrees of all animals were traced back to the founder populations and mean inbreeding coefficients per breed were calculated using the algorithm in [19].

### Detection of genomic regions with selection signatures

#### Integrated haplotype score (iHS) test

The iHS score is based on a ratio of extended haplotype homozygosities (EHH) associated with each allele. Thus, the iHS method requires information on the status of the ancestral and derived alleles for each SNP. Before computing iHS, the ancestral allele of all bovine SNPs was established from http://genome.jouy.inra.fr/downloads/Bovine_Ancestral_Allele/ [20]. The iHS score was computed for each autosomal SNP using the R package “rehh” [21].

Single-site iHS values were computed across the genome for each breed and averaged within non-overlapping windows of 500 kb across the genome resulting in a total of 5033 windows. The window size was adapted based on the extent of LD as described by Qanbari *et al.* [9]. The standardized iHS was calculated as:

1 $$ \mathrm{i}\mathrm{H}\mathrm{S}=\frac{ \ln \left(\frac{iH{H}_A}{iH{H}_D}\right)-E\left[ \ln \left(\frac{iH{H}_A}{iH{H}_D}\right)\right]}{SD\left[ \ln \left(\frac{iH{H}_A}{iH{H}_D}\right)\right]} $$

where *iHH*_*A*_ and *iHH*_*D*_ represent the integrated EHH score for ancestral and derived core alleles, respectively. Values of iHS were standardized so that they followed a standard normal distribution [6]. To calculate the P value at the genomic level, iHS scores for each SNP were further transformed as p_*iHS*_ = − log[1 − 2|*Φ*(*iHS*) − 0.5|], where *Φ*(*x*) represents the Gaussian cumulative distribution function (under neutrality) and *p*_*iHS*_ is the two sided *P*-value associated with the neutral hypothesis (*i.e.*, no selection) [22]. In order to control for false positives, the R package “fdrtool” [23] was used with its default options for “statistic = *p*-value”, which uses the empirical data below the 75th percentile to determine the null distribution of the test statistics. After false discovery rate (FDR) adjustment within a breed, the genome-wide significance level was equal to approximately 0.003.

#### Global F_ST_

To better understand the genetic divergence among all breeds, F_ST_ was calculated using the HierFstat R package [24] with the unbiased estimator proposed by Weir and Cockerham [25]. The negative F_ST_ values obtained for 24 800 SNPs were set to 0, since negative values have no biological interpretation [2]. Raw global F_ST_ values were ranked and used to identify regions under positive selection. The empirical *P*-value was calculated for each SNP as a proportion of the total number of SNPs [26, 27]. As in [28], the genome-wide significance level was set to 0.001. *i.e.*, only the top 0.1 % F_ST_ values were considered to represent a selection signature. Hence, no adjustment was made for multiple-testing for this statistic.

### Bioinformatics analyses

A gene was considered as being under selection if it overlapped with significant genomic windows based on iHS or if it contained an unexpectedly high proportion of highly differentiated SNPs based on F_ST_ values. Gene annotation was performed by exploiting the knowledge on UMD3.1 locations of genes from the NCBI (ftp://ftp.ncbi.nih.gov/genomes/Bos_taurus/mapview/seq_gene.md.gz). Because the annotation of the bovine genome is still incomplete, BioMart (www.ensembl.org/biomart) was used to determine the orthologous human gene ID for each gene detected. Enrichment analysis of these genes was performed using DAVID 6.7 by aligning the detected genes to human genes [29]. Functional annotations (Gene Ontology (GO) Biological Process, GO Cellular Component, GO Molecular Function and Kyoto Encyclopedia of Genes and Genomes (KEGG) Pathway) were assigned to genes using the functional annotation tool.

## Results

### Inbreeding coefficients per breed

Mean inbreeding coefficients of 0.0059, 0.0163, 0.0046, 0.0118, 0.0333, 0.0043 and 0.0106 were found for Angus, Belgian Blue, Charolais, Hereford, Holstein-Friesian, Limousin and Simmental breeds, respectively. In addition, genomic relationships were determined by calculating the Euclidean distances between alleles among all animals of the seven breeds analyzed (Figure S1 [See Additional file 2: Figure S1]). This figure shows that all the breeds can be clearly distinguished except Holstein and Friesian, for which animals were assigned to either of the two breeds based on their greatest breed proportion but many of the animals were actually crosses between Holstein and Friesian.

### iHS test

**Fig. 1:**
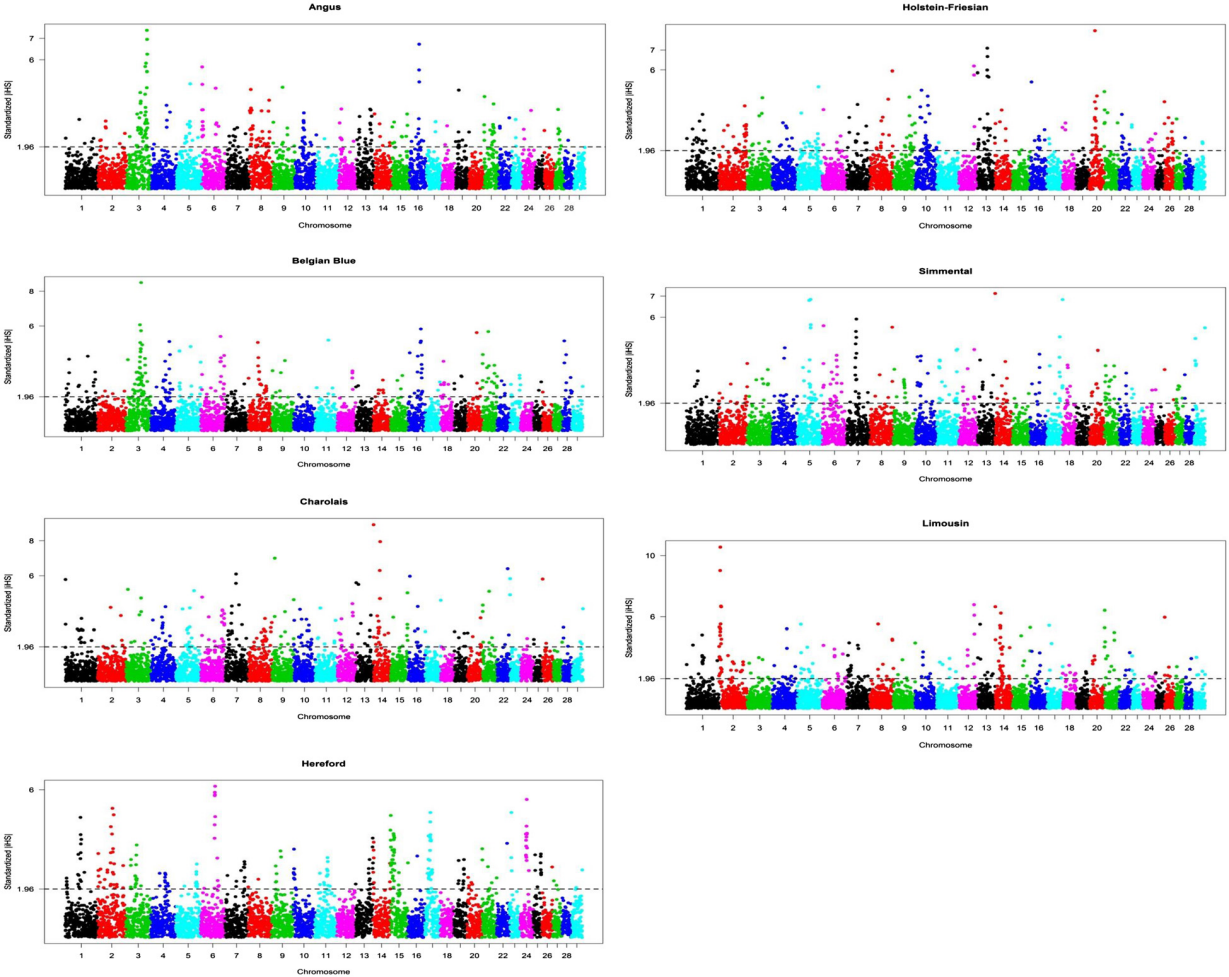
Genome-wide distribution of |iHS| values for seven bovine breeds

The 705 243 SNPs used in our study covered 2512.08 Mbp of the bovine genome (UMD3.1), with a mean distance of 3.56 kb between adjacent SNPs. The mean distance between adjacent SNPs per chromosome ranged from 3.41 kb on chromosome 25 to 3.81 kb on chromosome 13 [See Additional file: 1 Table S1]. Fig. 1 highlights the genome-wide distribution of |iHS| values to visualize the chromosomal distribution of selection signatures. After adjustment for FDR within each breed, 83, 92, 91, 101, 85, 101 and 86 signatures of selection were detected in Angus, Belgian Blue, Charolais, Hereford, Holstein-Friesian, Limousin and Simmental cattle, respectively. Selection signatures across the seven breeds were not uniformly distributed across the genome [See Additional file: 2 Figure S2]. No genomic region common to all breeds was detected.

A total of 434, 448, 543, 470, 466, 525 and 665 candidate genes overlapped with significant iHS genomic regions detected in the Angus, Belgian Blue, Charolais, Hereford, Holstein-Friesian, Limousin and Simmental cattle, respectively. Table 1 summarizes the genes that overlapped with the top five most significant iHS genomic regions per breed. These genomic regions harbored several candidate genes (full gene names of all gene symbols are in Table S2 [See Additional file: 1 Table S2]) including *SPATA6* and *FAAH* in Angus, *NEGR1*, *PIG*K and *RASAL2* in Belgian Blue, *SGK3* in Charolais, *SCFD2* and *SPATA18* in Hereford, *PRNP* and *PRND* in Holstein-Friesian, *BIN1* and *MSTN* in Limousin, and *SUOX* and *MMP19* in Simmental cattle. The other candidate genes are in Table S3 [See Additional file: 3 Table S3].

**Table 1:** Genomic region and associated genes of the top 5 significant iHS for each of the seven bovine breeds

Breeds	Genomic region (kb)	Mean |iHS| value	*P* value	Gene
Angus	Chr3:98500-99000	7.373	1.6 × 10^−13^	*SPATA6, SLC5A9, LOC100295301, SLINT1, LOC100337055, LCO787081*
	Chr3:99500-100000	6.953	3.6 × 10^−12^	*CMPK1, STIL, TAL1, PDZK1IP1, LOC100847677, LOC784358, CYP4A11, LOC784417, LOC787638, CYP4A22, LOC787656, CYP4B1*
	Chr16:46000-46500	6.726	1.7 × 10^−11^	*LCO513399, ERRFI1, PARK7, TNFRSF9, UTS2, LOC100848366, PER3, VAMP3, CAMTA1*
	Chr3:100000-100500	6.262	3.8 × 10^−10^	*KIAA0494, ATPAF1-AS1, ATPAF1, MOB3C, MKNK1, KNCN, DMBX1, LOC513210, FAAH, NSUN4, UQCRH, LOC100847274, LRRC41, RAD54L, POMGNT1, C3H1orf190, TSPAN1*
	Chr3:94000-94500	5.843	5.1 × 10^−9^	*ECHDC2, ZYG11A, ZYG11B, SELRC1, FAM159A, GPX7, ZCCHC11, LOC100138140*
Belgian Blue	Chr3:73000-73500	8.496	0	*NEGR1, LOC512165*
	Chr3:67500-68000	6.073	1.26 × 10^−9^	*AK5, PIGK, ST6GALNAC5*
	Chr16:61000-61500	5.833	5.44 × 10^−9^	*RASAL2,C16H1orf49*
	Chr21:29000-29500	5.681	1.34 × 10^−8^	*TJP1, TARSL2, TM2D3, LOC100335373*
	Chr88:96000-96500	5.401	6.61 × 10^−8^	*ANTXR2*
Charolais	Chr14:500-1000	8.913	0	*LOC781635, LOC100140130, LOC100848009, LOC784799*
	Chr14:33000-33500	7.945	2 × 10^−12^	*SGK3, C14H80rf45, LOC784087, LOC100847363, TCF24, PPP1R42, COPS5, CSPP1, APFGEF1*
	Chr9:13500-14000	7.000	2.56 × 10^−12^	*CD109, LOC100294729, LOC100336449*
	Chr22:50000-50500	6.401	1.54 × 10^−10^	*DOCK3, MAPKAPK3, CISH, HEMK1, C22H3orf18, CACNA2D2*
	Chr14:31000-31500	6.301	2.95 × 10^−9^	*CYP7B1*
Hereford	Chr6:70500-71000	6.142	8.15 × 10^−10^	*SCFD2, FIP1L1, LNX1*
	Chr6:69500-70000	5.893	3.79 × 10^−9^	*LRRC66, SGCB, LOC100335977, SPATA18, LOC100847183, USP46*
	Chr6:70000-70500	5.794	6.88 × 10^−9^	*USP46, MIR2445, LOC100847282, RASL11B, SCFD2*
	Chr6:69000-69500	5.760	8.43 × 10^−9^	*OCIAD1, OCIAD2, CWH43, DCUN1D4*
	Chr24:31500-32000	5.607	2.06 × 10^−8^	*ZNF521*
Holstein- Friesian	Chr13:47000-47500	7.088	1.36 × 10^−12^	*DIP2C, ZMYND11, PRNP, PRND, RASSF2*
	Chr13:48000-48500	6.670	2.55 × 10^−11^	*GPCPD1, LOC513580, LOC100140729, C13H20orf196, CHGB, TRMT6, MCM8*
	Chr12:73500-74000	6.194	5.88 × 10^−10^	*LOC100337129, LOC100299180, ABCC4, LOC530437*
	Chr13:46500-47000	5.991	2.08 × 10^−9^	*ADARB2, LOC100297660, WDR37, IDI1, GTPBP4, LARP4B, DIP2C*
	Chr8:108000-109000	5.951	2.67 × 10^−9^	*ASTN2*
Limousin	Chr2:5000-5500	9.019	0	*LOC507930, PROC, MAP3K2, ERCC3, CYP27C1, LOC784980, LOC524236, BIN1, MIR2350*
	Chr2:6000-6500	10.551	0	*HIBCH, C2H2orf88, LOC100335775, MSTN, OLC100335809, PMS1*
	Chr12:73000-73500	6.782	1.18 × 10^−11^	*LOC100849031, LOC528412*
	Chr2:9000-9500	6.685	2.31 × 10^−11^	*CALCRL*
	Chr14:500-1000	6.648	2.98 × 10^−11^	*LOC781635, LOC100140130, LOC100848009, LOC784799*
Simmental	Chr14:500-1000	7.124	1.05 × 10^−12^	*LOC781635, LOC100140130, LOC100848009, LOC784799*
	Chr5:64000-64500	6.847	7.55 × 10^−12^	*LOC100300928, UHRF1BP1L*
	Chr17:75000-75500	6.835	8.18 × 10^−12^	*DGCR8, TRMT2A, RANBP1, LOC526847, LOC100848428, RTN4R, LOC100336451, LOC100138815, LOC100301173, HSFY2, LOC786340, LOC100336511*
	Chr17:75000-475500	6.796	1.07 × 10^−11^	*ESYT1, ZC3H10, LOC100848780, PA2G4, ERBB3, RPS26, IKZF4, SUOX, RAB5B, CDK2, PMEL, DGKA, WIBG, LOC785991, MMP19, MGC142702, DNAJC14, ORMDL2, SARNP, GDF11, CD63, RDH5, BLOC1S1, ITGA7, METTL7B, LOC520938, OR10P1, OR6C4, LOC530539, LOC781363, LOC515967*
	Chr7:46500-46000	5.914	3.34 × 10^−9^	*FSTL4*

Further details are n Table S3 [See Additional file: 3 Table S3]

Table 2 summarizes the total numbers of overlapping candidate genomic regions between two breeds detected by iHS. Four significant genomic regions were common to both British breeds (*i.e.*, Angus and Hereford) and 17 genes [See Additional file: 3 Table S4] overlapped with these regions. In addition, 21 significant genomic regions were common to both continental breeds (*i.e.*, Charolais and Limousin) and 111 genes [See Additional file: 3 Table S4] overlapped with these regions. One genomic region was common to all four breeds and contained six genes [See Additional file: 3 Table S4]. Table S5 [See Additional file: 3 Table S5] summarizes the GO molecular function and biological process terms that were significantly enriched among the candidate genes in these putative regions under selection. These candidate genes were enriched in 33, 38, 45, 10, 71, 12 and 27 GO terms in Angus, Belgian Blue, Charolais, Hereford, Holstein-Friesian, Limousin and Simmental cattle, respectively. The GO terms were associated with fatty acid metabolism, reproductive traits, and both meat and milk production. Furthermore, all the candidate genes identified by iHS were overrepresented in the olfactory transduction and the PPAR signaling pathway (Table 3).

**Table 2:** Number of candidate genomic regions for each breed (on the diagonal) and number of overlapping candidate genomic regions between pair-wise breeds (below the diagonal) detected by iHS

Breed	Angus	Belgian Blue	Charolais	Hereford	Holstein-Friesian	Limousin	Simmental
Angus	83						
Belgian Blue	10	92					
Charolais	7	10	91				
Hereford	4	3	3	101			
Holstein-Friesian	2	8	14	5	85		
Limousin	6	7	21	8	15	101	
Simmental	7	4	15	3	4	11	86

**Table 3:** Enriched pathway terms for genes in regions under selection with count, ratio and *P*- value for seven bovine breeds

Breed	Pathway terms description	Count	*P* value
Angus	hsa03320:PPAR signaling pathway	5	0.0236
	hsa00071:Fatty acid metabolism	4	0.0252
Charolais	hsa04740:Olfactory transduction	20	0.0000
Hereford	hsa00310:Lysine degradation	4	0.0457
Holstein-Friesian	hsa04520:Adherens junction	5	0.0286
Limousin	hsa04514:Cell adhesion molecules (CAM)	7	0.0224
Simmental	hsa04740:Olfactory transduction	37	0.0000
All	hsa03320:PPAR signaling pathway	5	0.0445

### Global F_ST_

**Fig. 2:**
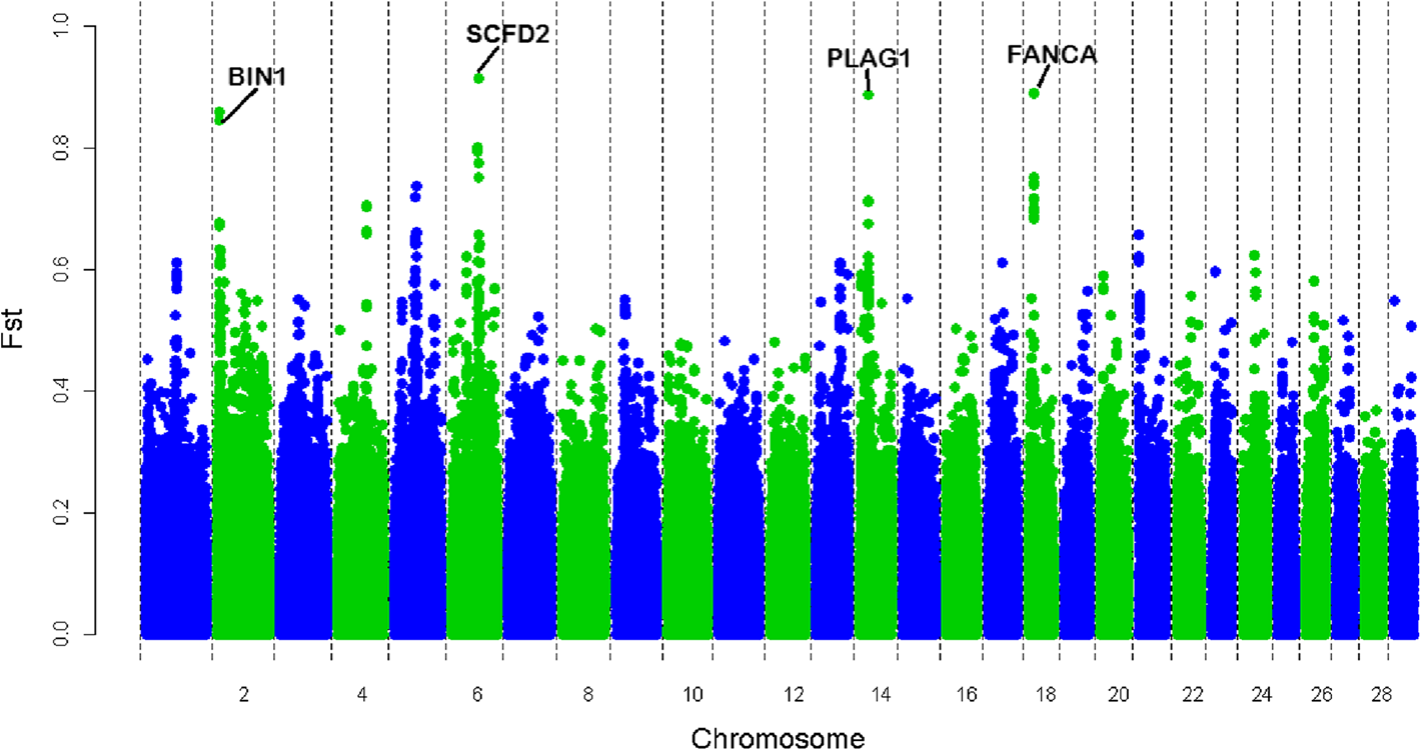
Genomic distribution of F_ST_ values

Several obvious genomic regions with high F_ST_ values were detected (Fig. 2). The mean genomic F_ST_ value across all SNPs was equal to 0.0876, indicating moderate genetic differentiation (F_ST_ ranged from 0.05 to 0.15) according to Wright’s classification [30]. One SNP had an F_ST_ value greater than 0.9, six SNPs had an F_ST_ between 0.8 and 0.9, 27 SNPs an F_ST_ between 0.7 and 0.8, 74 SNPs an F_ST_ between 0.6 and 0.7, and 294 SNPs an F_ST_ between 0.5 and 0.6. Four sharp F_ST_ peaks were clearly observed on chromosomes 2, 6, 14 and 18 (Fig. 2).

In total, 357 of the 704 significant F_ST_ values were located in 120 gene regions. The locus with the greatest F_ST_ value (0.914) was within an RNA region of the *SCFD2* gene [See Additional file: 3 Table S6]. Seventeen other SNPs with significant F_ST_ values also resided within this genomic region. The genes that overlapped with the three other F_ST_ peaks were *FANCA*, a candidate for breast cancer susceptibility [31], *PLAG1* that is associated with stature and body weight [32], and *BIN1* that is associated with Alzheimer’s disease [33]. Some of these 120 genes were overrepresented in the PPAR signaling pathway (Table 3). In addition, 13 GO terms that are associated with bone development, metabolic response and reproductive traits [See Additional file: 3 Table S5] were represented by these 120 genes.

### Genes that were detected by both methods

Table 4 lists the genes that were detected by both the iHS and F_ST_ approaches and that are located within or overlap with significant selection signatures. Ten, two, four, 17, 14, 11 and seven genes were detected by both methods in Angus, Belgian Blue, Charolais, Hereford, Holstein-Friesian, Limousin, and Simmental cattle, respectively. Of these genes, 12 were present in more than three breeds which included *DCUN1D4* and *OCIAD1*. In total, 57 unique candidate genes were identified by both the iHS and F_ST_ methods across the seven cattle breeds (Table 4).

**Table 4:** Common genes identified by the complementary approaches iHS and F_ST_

F_ST_	REHH	Genes
All breeds	Angus	*BPIFA4, BPIFB1, DCUN1D4, EPB41L1, LOC100337489, NECAB3, OCIAD1, PRDM5, SCP2, SNTA1*
	Belgian Blue	*LOC100848941, UBE3A*
	Charolais	*LOC530539, MGC142702, R3HDM1, TPRG1*
	Hereford	*DCUN1D4, FKBP5, FRYL, IMPACT, KCNN2, LNX1, LOC786242, LOC789547, LOC789558, MAML3, OCIAD1, PTK2, SCFD2, SGCB, SPATA18, TTC39C, ZNF521*
	Holstein-Friesian	*ACSF3, ANKRD11, C18H16orf7, CPNE7, FANCA, LOC100299071, LOC100848941, LYN, SPG7, TGS1, TMEM68, UBE3A, XKR4, ZNF276*
	Limousin	*BIN1, COL5A2, HERC2, INPP1, MFSD6, OSGEPL1, PLAG1, PMS1, SDR16C5, SLC40A1, TPRG1*
	Simmental	*CNPY2, DCUN1D4, KIT, LOC530539, MGC142702, OCIAD1, SCFD2*

## Discussion

In this work, our aim was to detect selection signatures in cattle using high-density genotypes (777 962 SNP) for seven breeds, with a mean distance between adjacent SNPs of 3.56 kb. Qanbari *et al.* [34] suggested that, in cattle, core regions may be more accurately detected by the relative EHH statistic if more than 50 000 SNPs were used. Barendse *et al*. [35] also suggested that more than 150 000 evenly spaced SNPs on the bovine genome would be an ideal number to accurately detect selection signatures using the F_ST_ approach.

Using simulations, Vilas *et al.* [36] recommended caution regarding the extent of false positive selection signatures which could be in fact false positive results. They detected apparent selection signatures on a large proportion of the simulated chromosomes for which actually no QTL had been simulated. In order to control the false positive rate in our study, we applied FDR adjustment within each breed. For F_ST_, only the top 0.1 % F_ST_ values were considered as representing selection signatures as recommended in previous studies [26–28]. Nonetheless, it is likely that some of the apparent selection signatures detected in our study may represent false positive results. However, since many of the selection signatures that we detected are consistent with other reports on selection signatures in independent cattle populations [9, 34, 37–41] and since, overall, they confirm the results of genome-wide association studies for the same traits [42–44], we consider that the number of false positive results in our study is small. Applying a stricter FDR would inevitably reduce the number of true positive selection signatures detected.

Identifying recent positive selection signatures in domesticated animals can provide information on genomic regions that are under the influence of both artificial and natural selection, and thus, can help the identification of beneficial mutations and underlying biological pathways for economically important traits. Here, we used two different, yet complementary, statistical approaches, iHS and global F_ST_, to detect selection signatures. The iHS approach appears to be the most powerful for detecting ongoing selection processes for which the target allele has a moderate to high frequency (0.4 < P < 0.8) within a population [45]. Once an allele becomes fixed, the iHS statistic can still identify selection signatures in the regions of strong LD surrounding the selected site rather than the region itself because fixation eliminates variability at and near the selected site. If the iHS method detects a genomic region, this region can contain several loci that may actually be undergoing selection within the breed. Therefore, the iHS method can detect breed-specific candidate genes under positive selection. For example, the *MC1R* gene that plays a role in coat color types, including black and white coats and spotted phenotypes, was only detected by the iHS analysis in the Holstein-Friesian population. Previously, this method was successfully applied in human [6, 46] and pig [47] populations as well as in other international cattle populations [9, 22, 48].

Global F_ST_ values are useful to detect selection signatures across breeds (*i.e.*, loci for which alleles are differentially fixed in different breeds) [16]. Global F_ST_ analysis identifies selection signatures that are common to different breeds, and determines how divergent selection may have affected the genomic pattern of these breeds. In our study, global F_ST_ analysis highlighted genes that are associated with phenotypes that differ among breeds, in particular, coat color and body size. It has been shown that the *KIT* gene is associated with the level of white coat spotting in cattle [49, 50], which differentiates the breeds included here. For the *PLAG1*, *LYN* and *TGS1* genes, several studies have documented their association with stature in both cattle and human populations [32, 51–54], thus, they may explain the differences in height between the breeds investigated in our study. One genomic region was detected by both iHS and global F_ST_ analyses, which indicates that both positive and divergent selection is acting on this region. Furthermore, complete concordance was found between the genes within regions of selection signatures identified by both methods (Table 4), which probably indicates true positive selection signatures. Integrating these two complementary approaches provides a valuable tool for positioning genomic regions that have undergone positive selection with more confidence.

Genotyping SNPs, which were discovered in another study, can result in ascertainment bias [55] and if the protocol used to identify the SNPs is not known, it will not be possible to directly correct for ascertainment bias. Based on simulations, Voight *et al.* [6] generated SNPs that had the same allele frequencies as in the real dataset in order to control, at least partially, for the effects of ascertainment bias. Nevertheless, in most studies, little or no attempt has been made to correct for ascertainment bias, and its effect is currently unknown. Previous genome-wide studies to detect positive selection in cattle have used the Bovine SNP50 BeadChip, which can also suffer from ascertainment bias due to the protocol used to discover the SNPs as well as to limited resolution. High-density SNP panels such as the Illumina BovineHD SNP chip have been designed to be less sensitive to ascertainment bias [56]. Furthermore, iHS analysis exploits information on allele frequencies of both selected and neighboring SNPs, which increases its power to detect selection signatures [1]. This method is more suited to genotyping data generated from SNP chips than to whole-genome sequence data, which minimizes the problems of ascertainment bias [34, 57]. To completely overcome ascertainment bias, a large-scale whole-genome sequencing project across breeds is necessary.

Although ascertainment bias may occur with the genotyping data used in our study, its effect is probably the same along the whole genome, unlike selection pressure that acts at certain genomic regions and will impact F_ST_ values for those regions only. The mean (± standard deviation) minor allele frequency (MAF) per SNP was similar in all breeds and ranged from 0.224 ± 0.162 (Angus) to 0.245 ± 0.158 (Hereford). In general, methods for the detection of selection signatures are designed to analyze non-related animals. Our animal sample consisted of 3122 dairy and beef animals from seven breeds. All animals were selected for genotyping with the high-density SNP panel to maximize imputation accuracy of their descendants from lower-density genotypes. Thus, although these animals were related, they were chosen to have as many progeny as possible and to be as genetically diverse as possible. Furthermore, they were highly selected animals and although it is likely that some false positive selection signatures may result from random genetic drift (and other factors), many of the detected signals probably reflect true selection signatures. Inbreeding will increase the extent of LD, which may result in false positives or type I errors. However the level of inbreeding in the populations analyzed here was relatively low.

Apart from inbreeding, the demographic history of a population can also influence the variome, i.e. the whole set of genetic variations found for a population of a given species, which complicates the interpretation of selection signatures. Expansion of a population increases the frequency of alleles that originally have a low frequency compared to expectations under a neutral model. Similarly, recent positive selection for an allele may have begun from a set of beneficial alleles with a higher initial frequency [20]. Such alleles may have been introgressed into a population through historical crossbreeding and, thus, be included in various haplotypes, which prevents LD-based estimators to detect the selection signature. Furthermore, crossbreeding can also generate false selection signatures, if for example a large conserved region of the genome from another breed is mixed with many smaller segments from the genome of the original breed [9].

Genome-wide analyses of selection signatures were reported for several international Holstein populations including Chinese [37], German [9, 34] and Israeli [40] Holsteins. Several of the genes that we identified here confirm previously documented selection signatures in Holstein cattle populations, such as *ACTC1* [9], *FABP3* [34], *RORA* [34], *GHR* [34] and *LACTB* [34]. Of particular interest is the region on BTA20 that was detected by the iHS method as having a strong selection signature in Holstein-Friesian cattle (Fig. 1) and [See Additional file: 2 Figure S2]. This result confirms selection signatures reported in a population of Israeli Holstein cows [40] and to a lesser extent in German Holstein cows [34]. This region on BTA20 between 20 and 40 Mb harbors many genes including *GDNF*, *WDR70*, *NUP155*, *GHR*, *ITGA2*, *LOC100847619*, *ITGA1*, *PELO*, *NDUFS4*, *FST*, *LOC100847646*, *LOC782165*, *MOCS2*, *ITGA2*, *NIM1*, *ZNF131*, *LOC100848437*, *LOC100336494*, *LOC785615*, *LOC785744*, *LOC100139184*, *LOC100848479*, *LOC783463*, *LOC527137*, *SEPP1*, *CCDC152*, *LOC100848533*, *PARP8*, *EMB* and *LOC785429*.

For the beef cattle populations studied here, several of the candidate genes found for body size were previously reported in horse [58], human [15, 53], dog [7] and/or cattle [39] populations. These genes included *CHCHD7*, *PLAG1* and *SMAD2* for the Limousin breed, *GDF5* for the Angus and Simmental breeds, *CDK6* for Simmental, *JAZF1* and *PRKG2* for Belgian Blue but selection signatures that overlapped with these genes were not found for the Holstein-Friesian population. Furthermore, some of the genes that overlapped with selection signatures in our study were consistent with those identified in other beef cattle populations [9, 38, 39, 41], such as *ACTC1* in the Charolais and Holstein-Friesian populations, a gene that is related to muscle formation [9].

In addition, some of the candidate genes that we detected were previously found by GWAS on cattle populations. Several of the genes that were identified here by the iHS analysis were previously suggested to be associated with milk production, fertility, body size or body conformation [42–44]. Apart from the aforementioned genes associated with body size, body weight and feed intake, we also identified *DGAT1*, *ABCG2*, *MSTN*, *GHR*, *CAPN3*, *PDGFRA*, *GAS1*, *ZNF521* and *TMEM130*. Biological justifications of why many of these genes reside within selection signatures were discussed in detail elsewhere [37]. Detection of selection signatures and GWAS are two different approaches to identify candidate genes of interest [59]. GWAS evaluates the relationship between genotype and phenotype, while detection of selection signatures relies on population genetic and evolutionary parameters that are obtained only from genomic information.

In our study, it should be noted that *DGAT1* and *ABCG2* were not found within a selection signature in the Holstein-Friesian population, whereas they were detected in the Limousin and Charolais populations. Both genes exhibited selection signatures only in the beef breeds and not in the dairy breed. Previously, *DGAT1* and *ABCG2* were detected in selection sweeps [39, 60] and by GWAS for performance traits [61] in cattle. The results of the present study are nonetheless consistent with the results of Kemper *et al.* [39] who detected selection signatures in Limousin and Charolais populations for *DGAT1* and *ABCG2*, but not in a Holstein population. The reason for not finding these genes in the Holstein-Friesian population used in our study may be that their alleles are no longer segregating in the population and therefore could not be detected by the iHS statistic. Possibly, the alleles that are still segregating, even after the intensive artificial selection during domestication, may have unfavorable pleiotropic effects that prevent their frequency from increasing in the Holstein-Friesian population. In addition, selection is likely to have affected standing variation. If the selected mutations were segregating on multiple different haplotypes before selection began, the iHS statistic may have too little power to detect the selection signature.

Some of the genomic regions that we identified here were previously documented to be under selection in human and other livestock populations. One selection signature region that was observed in the global F_ST_ analysis was on BTA 2 between 61881578 and 62129511 bp and contained the *R3HDM1* and *LCT* genes [62]. These two genes are associated with energy homeostasis; *R3HDM1* has a role in efficient food conversion and intramuscular fat content in some breeds [62, 63], while *LCT* is involved in the digestion of lactose in human adults [64]. These two genes have also been shown to be under positive selection in human populations [64]. In addition, some of the candidate genes (*TBC1D1*, *WIF1*, *LEMD3*, *KIT*, and *BMP2*) that we detected here were previously found within selection signatures in pig [65, 66], sheep [28, 67] and horse [68] populations.

It should also be noted that we detected several poorly annotated genomic regions that appear to have undergone strong selection. For example, genomic regions that had the greatest *P*-value estimated by the iHS method were on BTA20 in the Holstein-Friesian population [See Additional file: 2 Figure S2] but no genes in this region have been documented (Table 2). Similar patterns were also observed for F_ST_ signatures [See Additional file: 3 Table S6]. This observation is consistent with other genome-wide analyses of selection signatures in cattle [9], thoroughbred horses [68] and humans [6]. Thus, these results suggest that regions that do not appear to contain genes may also have an important role in adaptive evolution. Another reason, particularly in cattle, may be due to the relatively poor annotation of the bovine genome. Priority should be given to an improved annotation of the genomic regions that are suspected to be within positive selection signatures.

To better understand the molecular functions of these genes, we examined their GO classifications. Many of the genes detected in our study are consistent with expectations since they are involved in fatty acid metabolism, reproductive traits, and both meat and milk production. An intriguing candidate pathway that we identified is the PPAR signaling pathway which is known to be associated with meat quality and production traits in pigs [69] and cattle [44]. These observations need to be explored and verified in an independent population.

## Conclusions

We used two complementary methods (iHS and global F_ST_) to detect selection signatures across the whole bovine genome and across seven diverse cattle breeds using high-density genotypes. Our analyses revealed multiple genes under positive selection, which are related to milk production, reproduction, body size, muscle formation and coat color. Moreover, we identified the PPAR signaling pathway, which is an intriguing candidate pathway. Our results can contribute to the identification of the variants that underlie the detected selection signatures. In most cases, further studies are required to distinguish between selection signatures that are due to breed-specific characteristics or traits of practical interest for agriculture. However, both types of selection signatures are relevant to better understand the mechanisms and identify the targets of natural and artificial selection in domesticated cattle.

## Additional files

Additional file 1: Table S1. Distributions of SNPs after quality control and average distances between adjacent SNPs on autosomal chromosomes. **Table S1.** shows the total number of SNPs on each autosome and on all the autosomes, the length of each autosome and their total length and the average distances between adjacent SNPs on each autosome and for all the autosomes after quality control of SNPs. **Table S2.** List of full gene names for all gene symbols mentioned in this paper. Additional file 2: Figure S1. Population structure across eight bovine breeds**.** Figure S1 shows the population structure for each of the eight bovine breeds analyzed (Holstein and Friesian are treated as separate breeds) based on genomic relationships that were determined by calculating the Euclidean distances between alleles among all animals. The darker is the grey color, the stronger is the degree of genomic relationship. **Figure S2.** Genomic map of selection signatures detected by the iHS method for seven bovine breeds. Figure S2 shows the genomic distribution of regions that show selection signals detected by the iHS method for seven bovine breeds. The red rectangles are the genomic regions with selection signals. Additional file 3: Table S3. Gene annotation of genomic regions detected by iHS in seven bovine breeds. **Table S4.** List of genes within candidate genomic regions that are shared between Angus and Hereford, between Charolais and Limousin and among these four bovine breeds. **Table S5.** Results of the GO analysis. Table S5 shows the results of the GO analysis for all genes under selection by enrichment analysis. Enrichment analysis was performed using DAVID 6.7 by aligning the detected genes to human genes. Functional annotations (Gene Ontology (GO) Biological Process, GO Cellular Component, GO Molecular Function and KEGG Pathway) were assigned to genes using the functional annotation tool. Each sheet shows the results of the GO analysis for one bovine breed. **Table S6.** Gene annotation of loci detected with the F_ST_ approach. Table S6 shows the results.
