# Supplementary material for: Detection of selection signatures in dairy and beef cattle using high-density genomic information
Source: Genet Sel Evol. 2015 Jun 19;47(1):49. doi: 10.1186/s12711-015-0127-3 (PMC4472243; doi:10.1186/s12711-015-0127-3)

Figure S1. Population structure across the eight different breeds (Holstein and Friesian treated as separate breeds) where a darker colour represents a stronger degree of genomic relationship


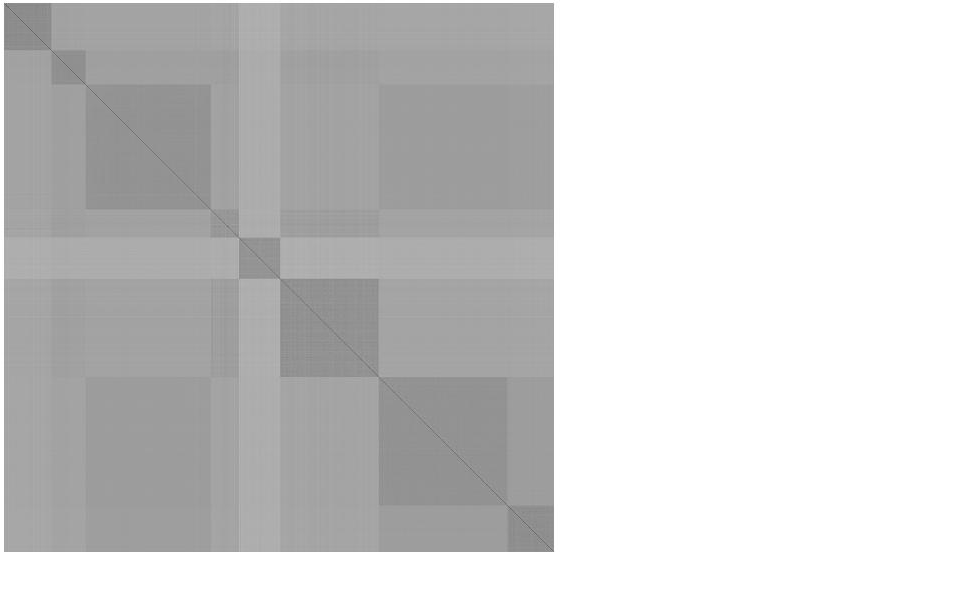


**Belgian Blue**

**Holstein-Friesian**

**Limousin**

**Hereford**

**Charolais**

**Simmental**

**Angus**

Figure S2. Genomic map of selection signatures detected by the iHS method for seven bovine breeds


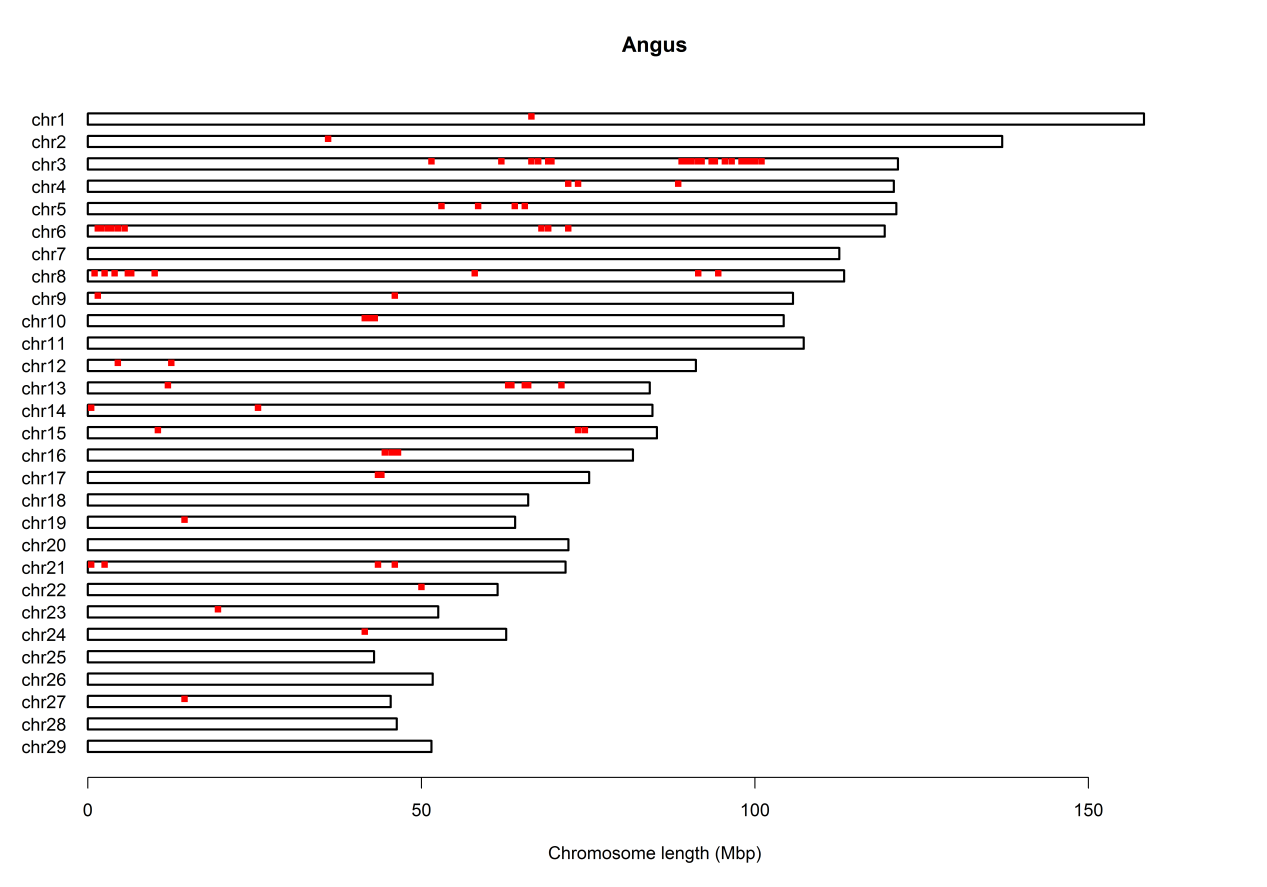

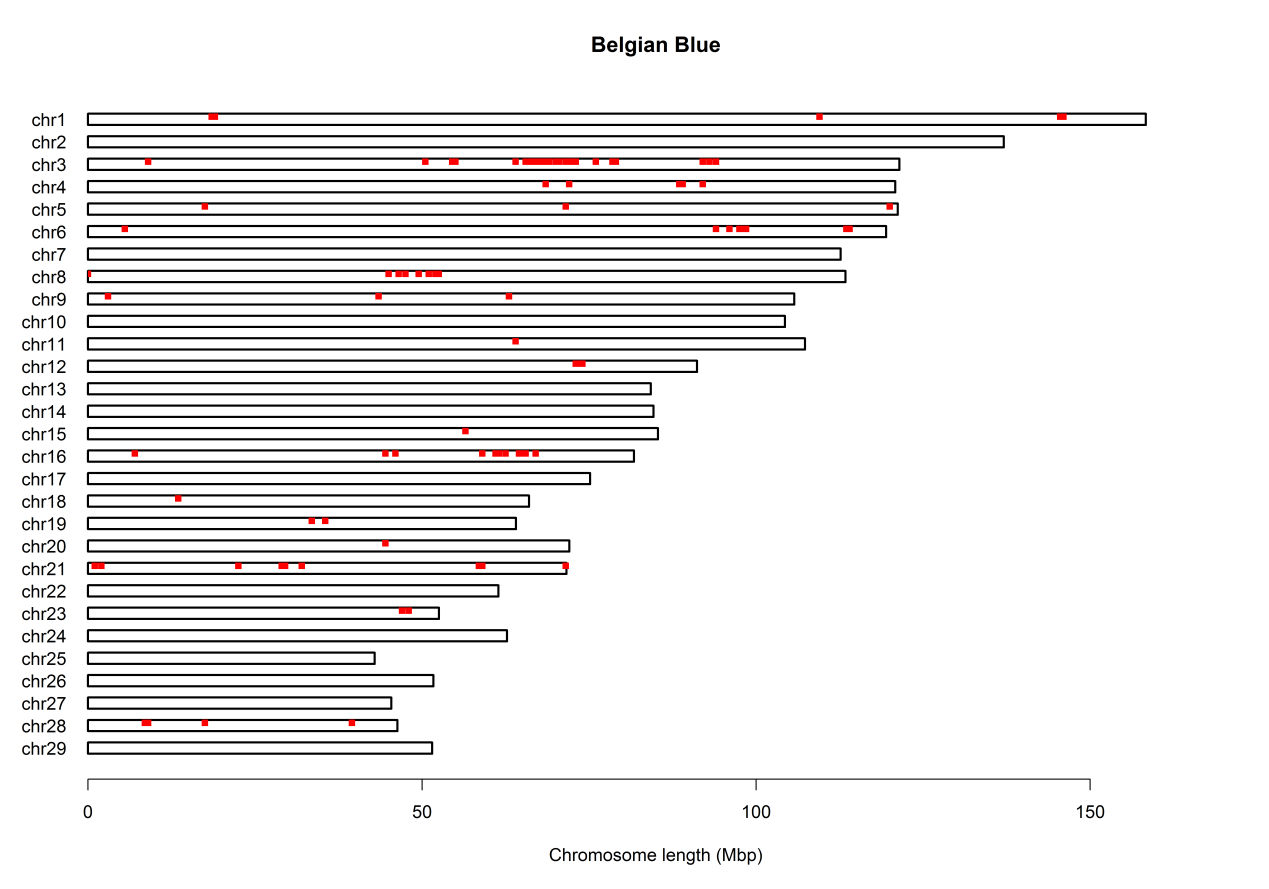

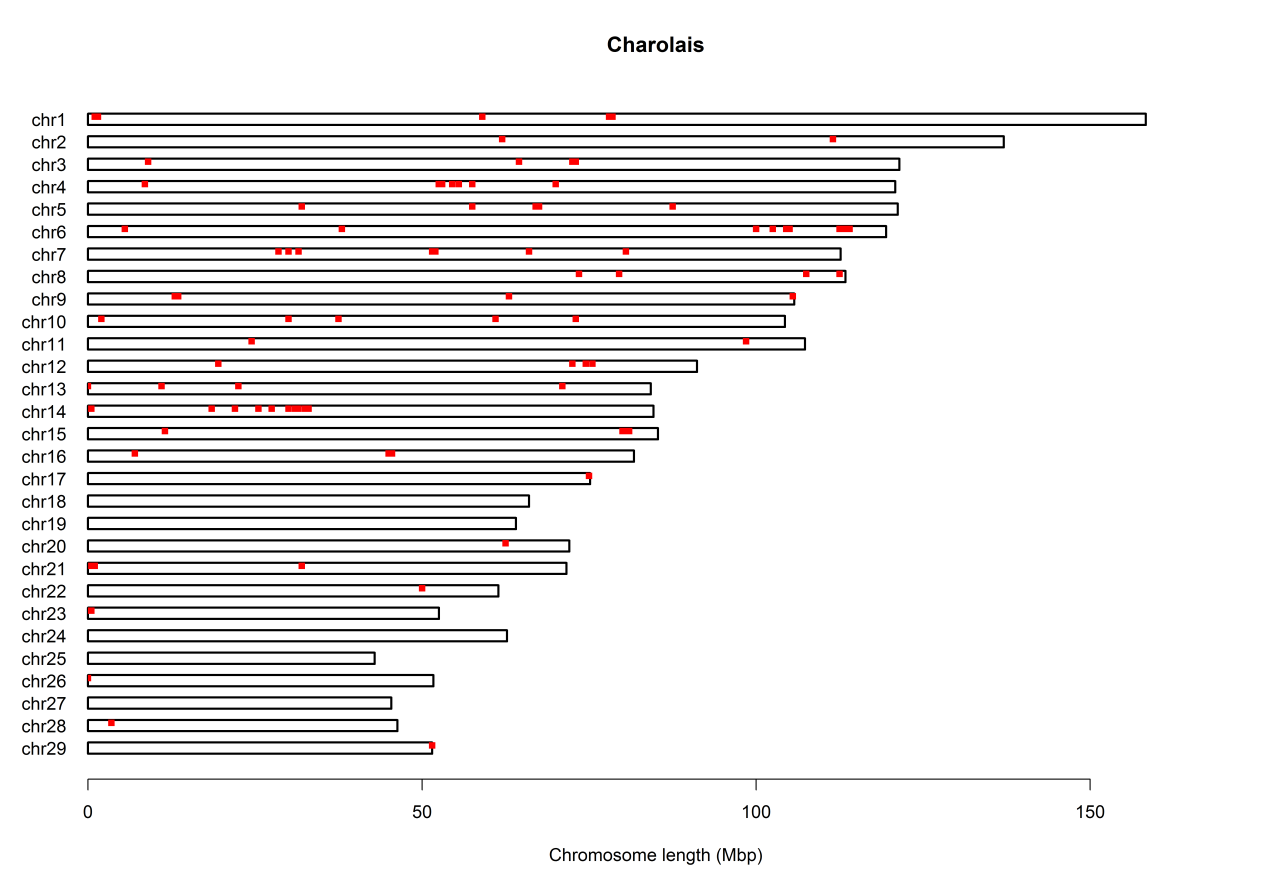

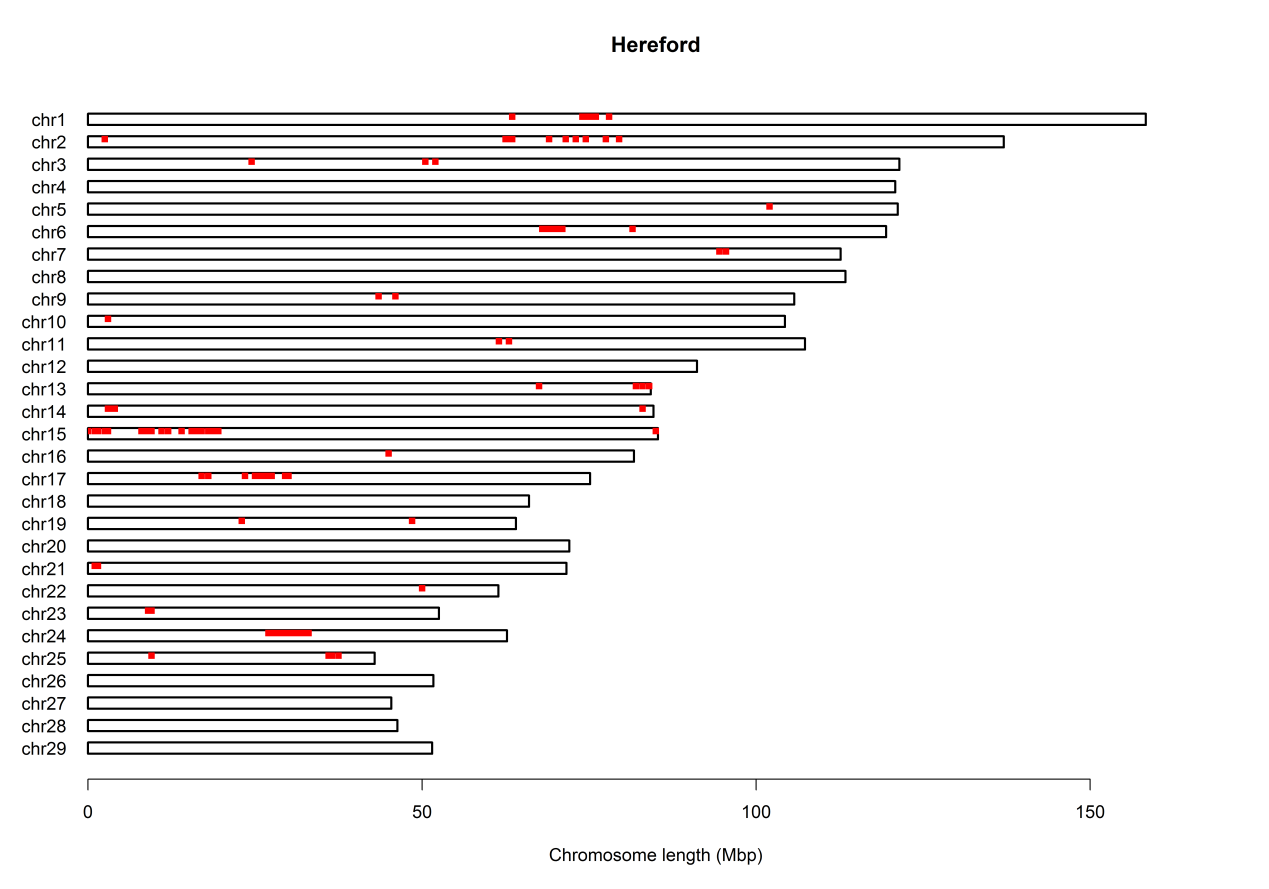

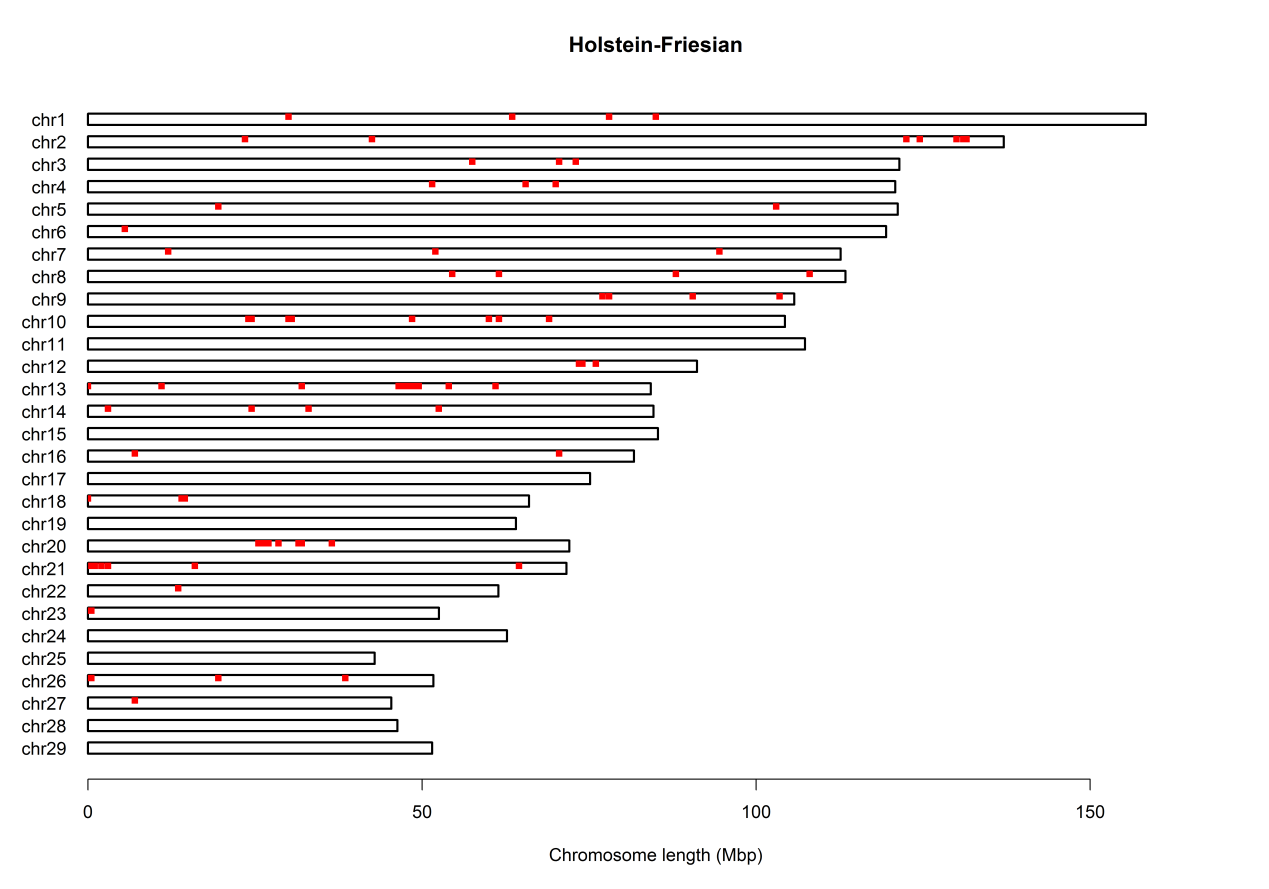

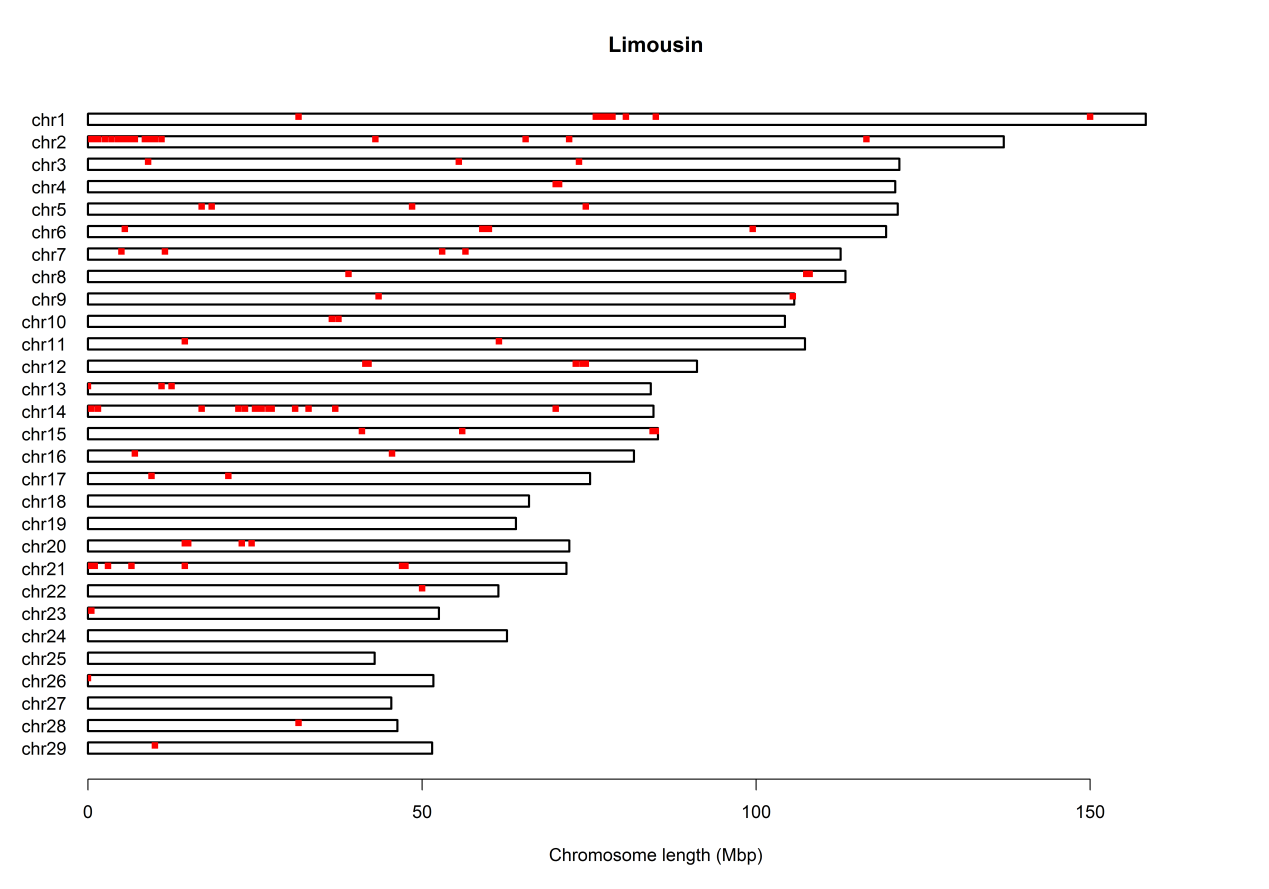

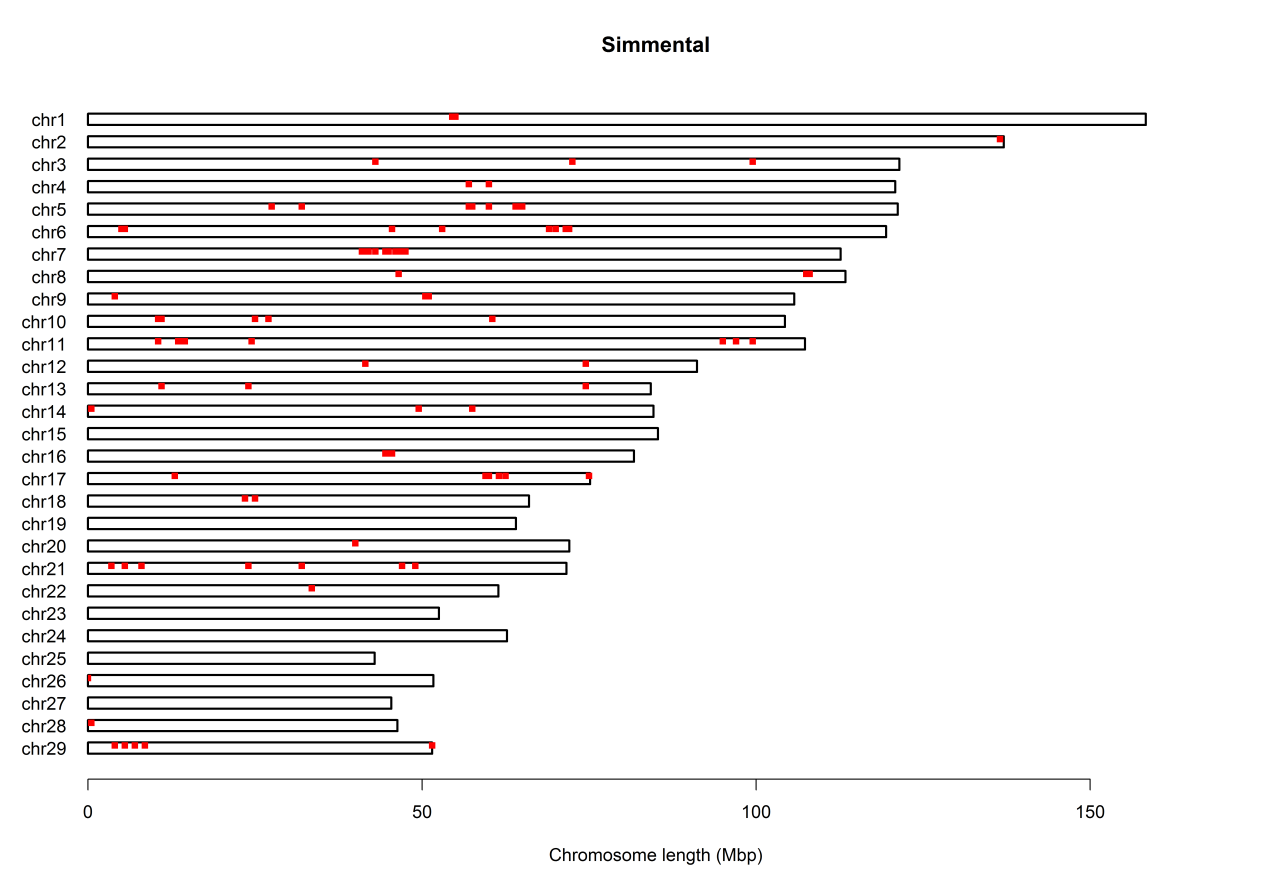

Supplement: Additional file 2: Figure S1. — Population structure across eight bovine breeds. Figure S1 shows the population structure for each of the eight bovine breeds analyzed (Holstein and Friesian are treated as separate breeds) based on genomic relationships that were determined by calculating the Euclidean distances between alleles among all animals. The darker is the grey color, the stronger is the degree of genomic relationship. Figure S2. Genomic map of selection signatures detected by the iHS method for seven bovine breeds. Figure S2 shows the genomic distribution of regions that show selection signals detected by the iHS method for seven bovine breeds. The red rectangles are the genomic regions with selection signals. [file 12711_2015_127_MOESM2_ESM.docx]
